# Photoinduced Plasmon‐Polariton Mediated Energy Storage

**DOI:** 10.1002/nap2.70313

**Published:** 2026-10-16

**Authors:** Nitzan Shauloff, Yury Turkulets, Ilan Shalish, Yossi Abulafia, Zeev Zalevsky, Raz Jelinek

**Affiliations:** ^1^ Department of Chemistry Ben Gurion University of the Negev Beer Sheva Israel; ^2^ Ilse Katz Institute for Nanotechnology Ben Gurion University. of the Negev Beer Sheva Israel; ^3^ Faculty of Engineering Sciences School of Electrical and Computer Engineering Ben Gurion University. of the Negev Beer Sheva Israel; ^4^ Materials Science & Engineering University of Michigan Ann Arbor MI USA; ^5^ Bar‐Ilan Institute for Nanotechnology & Advanced Materials Ramat‐Gan Israel; ^6^ Faculty of Engineering Bar‐Ilan University Ramat‐Gan Israel

**Keywords:** nano antennas, nano‐capacitors, plasmonic

## Abstract

Development of new means of remotely induced energy storage is important for varied applications, including minimization of energy loss and heat effects in computation devices, solar energy generation, and data transmission. Here, we demonstrate a new concept by which photoinduced energy storage is facilitated through plasmon‐polariton generation, propagation, and supercapacitor charging. Specifically, we designed a simple nano‐device, allowing conversion of photonic energy into plasmons, transmitting the plasmons over micrometer‐scale distances, and storing the energy in nano‐supercapacitors. The new platform may potentially minimize heat‐associated energy loss in computer circuitry and data processors, as energy is transmitted optically rather than electronically and thus it does not experience electrical resistivity. Moreover, the concept of photoinduced plasmonic mode that serves as an intermediary for electromagnetic energy transfer that charges supercapacitor, may theoretically be used to form the basis for a new “plasmonic solar cell” technology comprising simple and readily fabricated in‐plane modules which do not require expensive semiconductor materials or complex fabrication schemes encountered in photovoltaic cells.

## Graphical abstract

A novel concept of photo plasmonic voltaic energy storage captured in special nanocapacitor is presented. The photoinduced plasmon‐mediated capacitor is a basis for innovative “plasmonic solar cells” which could be manufactured from inexpensive plasmonic generating metals coated with readily available dielectric materials while being a surface fabricated device that does not require volumetric photonic energy conversion via its absorption in semiconducting material.

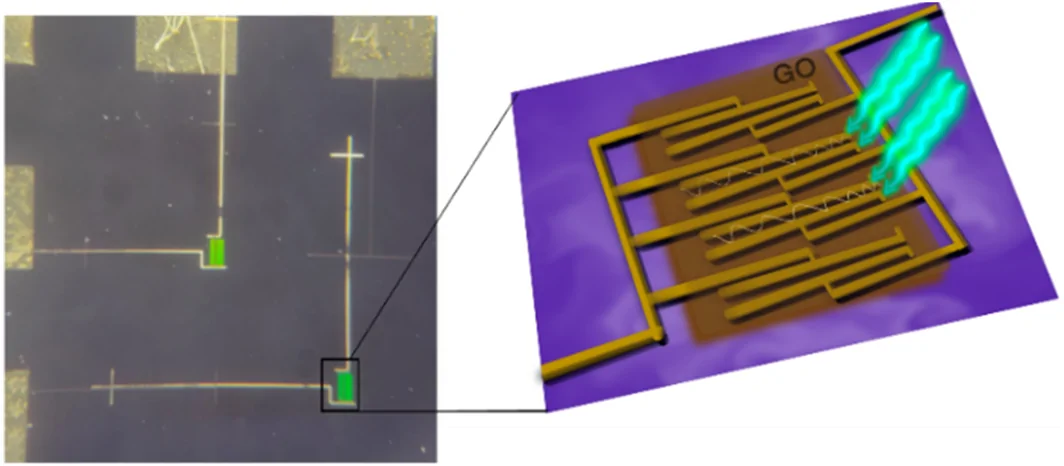

## 1 Introduction

The central processing units (CPU) and graphic processing unit (GPU) are the most power‐consuming parts in computers. The transmission of energy to/from millions (or billions) of transistors in a computer generates significant heat primarily due to current passing through the transistors and wires. This is problematic because part of the electrical energy driving the CPU and GPU is lost to heat, while sufficient cooling needs to be applied to prevent heat damage to the computer's internal circuits. These two intertwined limitations are ever more critical as computing demands are becoming ever more pronounced considering the rapid advances in data storage requirements, cloud computing, virtual currency mining, data‐centers etc. Indeed, energy heat loss in computer systems has been estimated to generate huge costs and increased carbon footprint.

This work introduces a powerful and innovative concept which may potentially lead to significantly minimized heat‐associated energy loss in computer circuitry and data processors, and, furthermore, may accomplish a new means for remotely implemented energy storage. Specifically, we demonstrate integration of wireless transmission of energy with its storage in a nano‐supercapacitor via photonic‐to‐plasmonic conversion and propagation. A key constituent in the novel system we present is a recently developed, unique nanophotonic element, termed *plasmonic antenna (plantenna)*, which efficiently converts light into plasmons (surface currents) [1]. The *plantenna* technology relies on nanometric antenna design matched for efficient operability at ultra‐high frequencies (photonic frequencies). The efficient conversion of light frequency into plasmons (essentially surface plasmons polaritons, SPPs) is facilitated through concentrating photon energy and mode matching between the photons and the SPPs, achieved via a metallic rig located in the center of the *plantenna* receiver [1]. Importantly, photon‐to‐plasmon conversion via *plantenna* design was demonstrated also in wireless connectivity between microelectronic devices [2]. Pseudo and super‐capacitors were proven to be good candidate for electrochemical energy storage [3, 4]. Specifically, in‐plane (surface fabricated) micro‐supercapacitors (MSCs) have garnered particular interest in microelectronics. Notably, as MSCs can be directly fabricated on microelectronics boards, they serve as an alternative energy source to traditional Li‐ion based micro‐batteries. The most efficient in‐plane MSC architecture design is the inter‐digitized electrode [5] which enables short ion diffusion path [6] resulting in excellent cycle stability and power density. A new Au‐coating method has been employed to construct highly porous pseudo capacitor‐coated electrodes [7, 8], as well as high energy density MSCs comprising hollow nickel microfiber network [9].

In this work, we constructed a simple nanodevice capable of light‐induced energy storage via photogenerated surface plasmons. Specifically, the system comprises an array of *plantennas* generating plasmons upon light irradiation, propagating the plasmon mode over distance, and subsequent charging with this energy a simple nano‐supercapacitor. The innovative concept may potentially facilitate future substitute to photo‐voltaic cells where the proposed concept will be generated on the outer surface of objects that are exposed to sun radiation and will convert this energy to the electric domain and store it.

It is important to note that plasmons are collective oscillations of the electron density relative to the positive ionic background and do not constitute free charge carriers. Likewise, surface plasmon polaritons transport electromagnetic energy and momentum rather than net electric charge. Our intention is not to suggest that plasmons themselves directly transport free charge to charge or discharge a capacitor. Instead, the plasmonic mode serves as an intermediary for electromagnetic energy transfer and field enhancement within the device. The resulting capacitor charging/discharging mechanism arises from the induced electronic response of its structure rather than from the transport of net charge by the plasmon itself.

## 2 Fabrication and Experimental Characterization

### 2.1 Plantenna Device Fabrication

The *plantenna* was fabricated by electron beam lithography (EBL), ion beam sputtering (Au, 20 nm) and lift off. SiO_2_/Si sample was spin‐coated with bi‐layer poly‐(methyl methacrylate) (PMMA 50K A6, PMMA 200K A4) electron‐beam resist providing thickness of 200 nm. The samples coated with PMMA were subsequently baked for 120 s on a hot plate at 180C. The desired pattern was exposed in the PMMA layer using a CRESTEC CABLE‐9000C high‐resolution electron‐beam lithography system using different doses to control line and gap width. Then the samples were developed for 40 s using methyl isobutyl ketone (MIBK) and rinsed with IPA. The samples were subsequently exposed to Ar plasma to etch 10 nm in order to remove leftovers from the pattern. Then the sample was sputter coated using BESTEC 2″ DC magnetron to deposit 5 nm Cr and 35 nm Au, and then immersed in 180 KHz ultrasonic bath with NMP for 3h for resist liftoff.

### 2.2 Preparation of GO Sheets

The preparation of large‐area graphene oxide (GO) sheets was carried out according to the following procedure: 2 g of natural flake graphite (average particle size: 500–600 μm), 2 g of NaNO_3_, and 96 mL of concentrated H_2_SO_4_ were mixed at 0°C under continuous magnetic stirring. Subsequently, 12 g of KMnO_4_ was gradually added while maintaining the temperature at 0°C. The resulting mixture was stirred for 90 min at 0°C and then heated to 35°C for an additional 2 h. To dilute the reaction mixture, 80 mL of distilled water was slowly added dropwise over approximately 30 min. This was followed by the addition of 200 mL distilled water and 10 mL of 30% H_2_O_2_, and stirring continued for a further 10 min, yielding a graphite oxide suspension. During this step, hydrogen peroxide reduced residual permanganate and manganese dioxide species to soluble manganese sulfate.

The graphite oxide was isolated by centrifugation at 16,000 rpm for 10 min and repeatedly washed with distilled water until a neutral pH was reached. The resulting material was then subjected to mild sonication (80 W, 5 min) to exfoliate the graphite oxide and produce a GO suspension. To obtain uniform large‐area GO sheets, the suspension was first centrifuged at 3000 rpm for 3–5 min to remove thick multilayer flakes and visible particulates. The collected supernatant was subsequently centrifuged at 5000 rpm for 5 min, enabling separation of large GO flakes (precipitate) from smaller flakes (supernatant). The precipitated large‐area GO sheets were then redispersed in distilled water to obtain a stable suspension. The process yield was determined by drying the collected large‐flake precipitate in a vacuum oven at 90°C for 24 h to completely remove residual water. The final mass of the recovered large‐area GO sheets was approximately 0.25 g, corresponding to an initial graphite loading of 2 g.

### 2.3 Electrochemical Measurements

The electrochemical performance of the synthesized graphene oxide electrode was evaluated using a three‐electrode configuration connected to a Biologic Autolab electrochemical workstation. The working electrode was prepared by dispersing 6 mg of graphene oxide in a mixture of 400 μL ethanol, followed by ultrasonication to obtain homogeneous ink. The resulting suspension was drop‐cast onto a 3 mm glassy carbon electrode (GCE). A platinum wire and Ag/AgCl electrode served as the counter and reference electrodes, respectively. All electrochemical measurements were conducted in 1 M Na_2_SO_4_ aqueous electrolyte. Cyclic voltammetry (CV) measurements were performed within a potential window of − 0.3 to 0.7 V at scan rates ranging from 10 to 200 mV s^−1^. Galvanostatic charge‐discharge (GCD) experiments were carried out at current densities of 1, 2, 3, 5, and 7 A g^−1^. Electrochemical impedance spectroscopy (EIS) measurements were conducted using an AC perturbation amplitude of 10 mV over a frequency range from 0.01 Hz to 100 kHz.

### 2.4 Supercapacitor Fabrication

1 g of graphite flakes were added to a round bottom flask containing 46 mL of H_2_SO_4_ cooled in an ice bath. The mixture was then sonicated for 1 h to disperse the graphite. After shifting the flask to a water bath, 1 g of NaNO_3_ and 6 g of KMnO_4_ were successively added very slowly (within 15 min). The temperature of the water bath was increased to 40°C and the reaction mixture was stirred for 3 h. 50 mL of deionized water was slowly added to the reaction mixture, followed by 5 mL H_2_O_2_. Stirring was continued for another 10 min. The reaction mixture was then centrifuged (5000 rpm, 20 min) to separate the precipitate. Centrifugation was repeated until the supernatant showed a neutral pH. Resulting brown colored residue of graphene oxide was then dried through lyophilization and stored at 4°C. GO was re‐dissolved in doubly distilled water. The solution was ultrasonicated for 1 h to obtain stable suspensions in a sonication bath at 37 KHz. The dispersion of GO and its derivatives (2 μL) were drop‐cast on the active area of the *plantenna* device and slowly dried at room temperature for an hour. The obtained *plantenna/GO device* were kept under nitrogen environment.

### 2.5 Characterization

*Scanning electron microscopy* (SEM)—Scanning electron microscopy (SEM) images of the deposited GO material on the *plantenna* device were acquired on a FEI Verios scanning electron microscope (Thermo Fisher Scientific, XHR 460L). The images were viewed at an acceleration voltage of 50 kV with different magnification.

### 2.6 Photovoltage Measurements

Surface photovoltage spectra (SPS) were acquired on the *plantenna/GO* device having a ground contact on their periphery. The photovoltage signal was acquired using a Besocke Delta Phi Gmbh Kelvin probe from samples placed in a dark Faraday cage at room temperature. The samples were illuminated using a 300 W Xe light source monochromatized by Newport Corp. MS257 monochromator. To prevent illumination by second order diffractions of the grating, the monochromatized light was further filtered by order‐sorting long pass filters. The photon flux was kept constant throughout the entire spectral acquisition by means of a variable slit operated in closed control loop.

## 3 Results and Discussion

### 3.1 The Plantenna Device

Figure 1 illustrates the photoinduced plasmon‐mediated energy storage nano‐device. An array of “plasmonic antenna” (*plantenna*) comprising nanofabricated 500 nanoscale antennae, with pitch of 800 nm (distance between adjacent devices) is fabricated on a silicon dioxide substrate. Each *plantenna* is basically an antenna that consists of two parallel metallic (Au) nanowire of 50 μm length separated by a 60−70 nm gap, with/without additional nano rod, termed director, is placed at proximity of only 30–40 nm to the dipole to enhance the antenna performance [1, 2]. The whole array was connected to biased metal lines directly connected to the 50 μm nanowires. The *plantenna* array is coated through drop casting with a graphene oxide (GO), serving as the dielectric medium, thereby forming nanocapacitors. GO has been widely employed in varied capacitor and supercapacitor configurations, taking advantage of its high surface area and abundant hydroxyl moieties which suppress proton conductivity [10, 11, 12].

**FIGURE 1:**
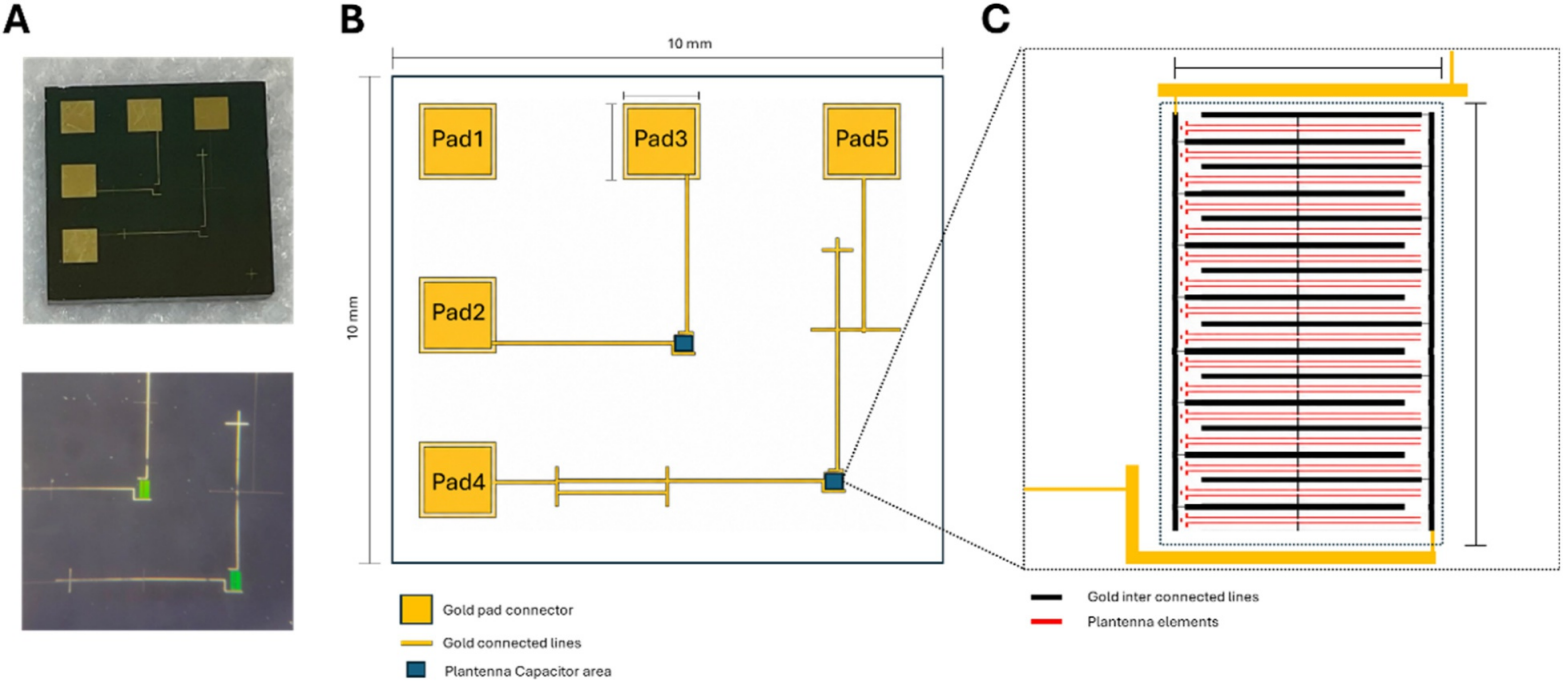
Optical micrographs and schematic representation of the fabricated chip architecture. (A) Optical images of the fabricated device showing the full chip layout and enlarged views of the interconnection regions. (B) Schematic overview of the chip consisting of five Au contact pads (Pad1–Pad5), conductive traces, and integrated sensing/interconnection regions. The chip dimensions were about 10 × 10 mm, while the individual contact pads measured about 1500 × 1500 μm. (C) Enlarged schematic illustration of the internal antenna array structure comprising the plantenna elements. The plantenna elements (red) were positioned between alternating ground fingers (black), where adjacent ground fingers were connected alternately to the upper and lower ground lines. All plantenna elements were interconnected through a central conductive line. The interspace between conductive figures (black) as well as their width was 10 μm. Gold (Au) was used as the conductive layer on a Si/SiO_2_ substrate.

The specific dimensionalities of the *plantenna* and the designed chip (as seen in Figure 1) were designed to achieve optimal light‐generated plasmons. Specifically, we targeted light in the green (around 530 nm) region of the spectrum, previously shown to exhibit the best plasmon‐generating efficiency [1, 2]. The photons after being converted to plasmons are propagated (the radiation propagates) along the metallic plasmonic waveguide while coupled and charging the nano capacitor. In Figure 2 we present the actual fabrication of the device that was schematically described in Figure 1. The figure depicts a representative scanning electron microscopy (SEM) image of the *plantenna*/nanocapacitor device. Overall, the device allows remote charging of the nanocapacitor through light irradiation of the *plantenna*: the *plantenna* device has two main parts. Its upper edge having the metallic rig and its main body containing two metallic wires. The edge with the rig aims to convert photons to plasmons. The two wires are waveguides and the plasmonic mode is guided along them. The mode is propagated on the interface between the metal and the dielectric material while the tails of the propagated plasmonic mode interacts with its environment. The nano capacitor positioned just above those two wires and thus the propagated plasmonic mode interacts with those nano dots and charges them.

**FIGURE 2:**
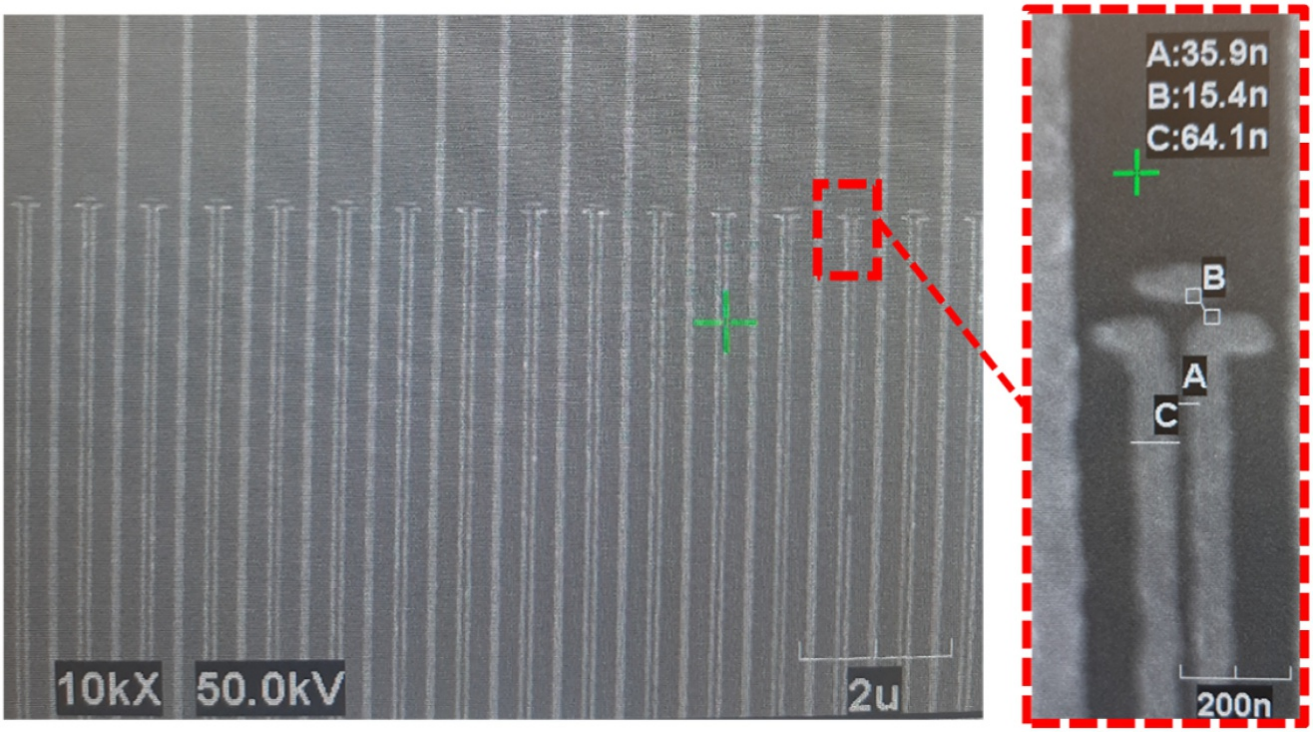
SEM image of the fabricated nano‐plantenna array, showing the periodic arrangement of the gold nanoconductors and antenna elements. The red dashed box indicates the region displayed at higher magnification in the inset, where representative nanoscale dimensions are marked. Scale bars correspond to 2 μm in the main image and 200 nm in the inset.

Figure 3 presents photoinduced voltage induced in the *plantenna*/nanocapacitor device measured by a scanning Kelvin probe. The Kelvin probe setup is illustrated in Figure 3A. The semitransparent Kelvin probe device facilitates recording of surface photovoltage spectra (SPS) under illumination, on a nanometer length‐scale and in a wide wavelength range. Specifically, the sample was held perpendicular to the probe and electrically connected to form a parallel plate capacitor. The probe is from a different material to the sample and thus has a different and distinct Fermi level. When electrical connection is made between the probe and the sample electron flow can occur between the probe and the sample in the direction of the lower to the higher Fermi levels, ultimately equilibrating the Fermi levels of the probe and sample. Furthermore, a surface charge develops on the probe and the sample, with a related potential difference. The probe vibrates along a perpendicular to the plane of the sample, and this vibration causes a change in probe to sample distance, which in turn results in the flow of current, taking the form of an AC sine wave. Note that under illumination which pass throw the GO capacitor, there is a generation of plasmonic waves (due to the *plantenna* devices), causing a change in the potential between the probe to the surface at a given electric field.

**FIGURE 3:**
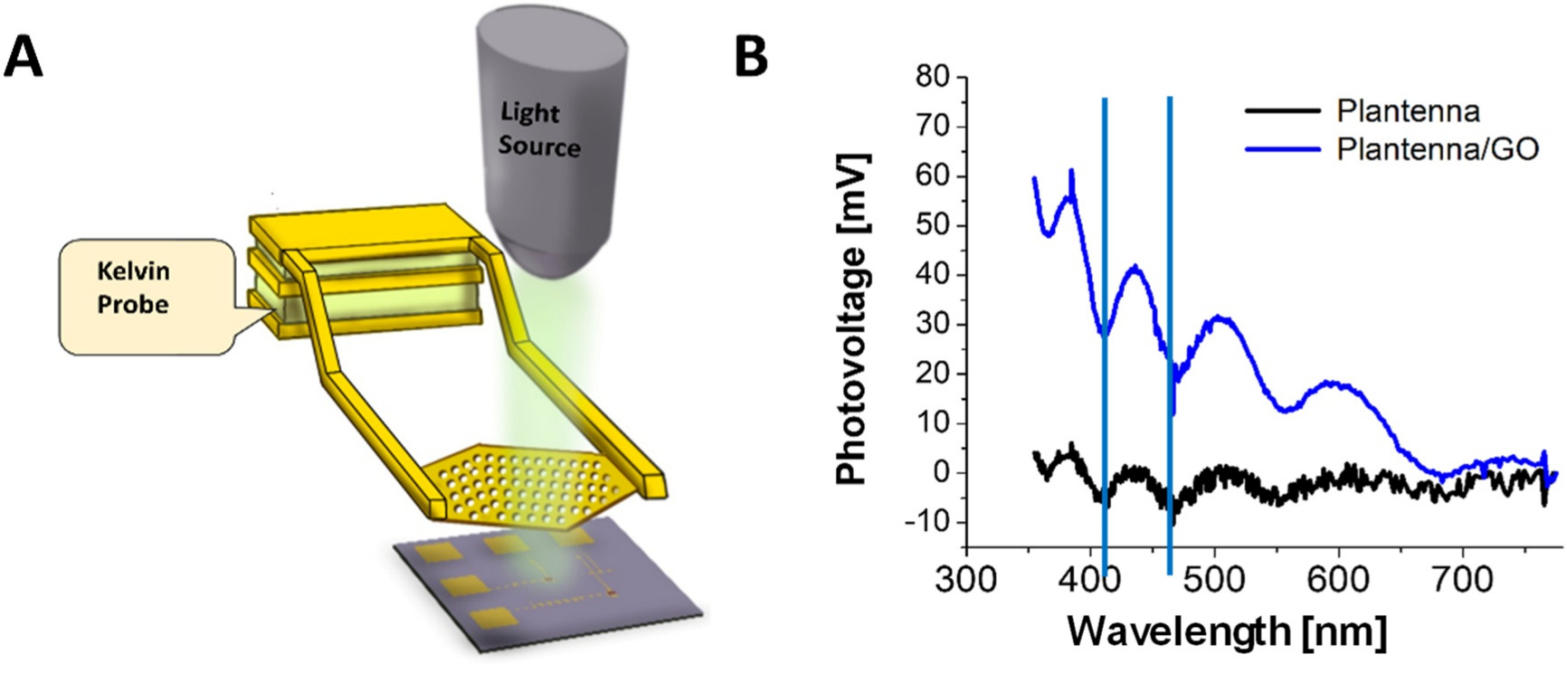
Investigation of the plantenna/GO spectral response using a Kelvin probe. (A) The setup for Surface Photovoltage spectroscopy measurement using a Kelvin probe in which the fabricated chips were tested and experimentally characterized. (B) The photovoltage response of the uncoated plantenna (black), and the plantenna/GO (blue) under illumination using the SPS method between 350 and 775 nm.

Figure 3B depicts the surface photovoltage recorded in the GO‐coated *plantenna* between 350 and 775 nm using the Kelvin probe apparatus. Importantly, more pronounced photovoltage was recorded in the GO‐coated electrode versus a control device that was not coated with the dielectric layer (blue spectrum vs. black spectrum in Figure 3B). As apparent in Figure 3B, the spectral responsivity is periodic. Indeed, due to the high uniformity of the fabricated surface a Fabry–Perot (F‐P) resonator might have been generated in the silica (SiO_2_) layer on top of which the metallic *plantennas* were fabricated (the *plantenna*'s metallic structure acted as one mirror and the second reflection was done from the interface between the SiO_2_ layer and the silicon wafer on top of which the SiO_2_ layer is positioned).

In F‐P resonators the distance between spectral peaks (constructive and distractive interference) in frequency domain (Δν) equals to:

(1) $$\Delta \nu =\frac{c}{2nL},$$

where *c* is the speed of light, *n* the refraction index of the medium between the two resonating mirrors and *L* is the size of the resonator (distance between the mirrors). The spectral periodicity in terms of wavelength domain (Δλ) equals to:

(2) $$\Delta \lambda =\frac{\lambda^{2}}{c}\Delta \nu =\frac{\lambda^{2}}{2nL},$$

After substituting *n* = 1.5 and *L* = 2 μm (The actual width of the SiO_2_ layer in the fabricated chips), we get for average wavelength λ of 450 nm (the mid of the measured spectral range), spectral periodicity of about Δλ = 34 nm which is close to the periodicity seen in Figure 3. Possible deviations could be related to the illumination angle which was not exactly perpendicular to the device's surface as assumed in Equations (1) and (2) (and indeed in the experimental validation we saw that the obtained spectral periodicity was highly angle dependent).

We characterized the optical response in a specific region on the device as well as examined the time‐resolved photovoltage response of the device, as presented in Figure 4. With optically initiating the region containing the *plantennas* (region 1), highlighted as the area where conversion of photons to plasmons occurs, we witness a photovoltage response. With comparison to the illumination on the region where no such conversion occurs (region two on the device) there was no photovoltage response observed. Importantly, only illumination in the exposed antenna region allowed energy transfer through plasmon generation and development of voltage on the surface. In contrast, direct illumination on the capacitor medium‐coated region did not yield photovoltage, since plasmons could not be produced in the electrodes.

**FIGURE 4:**
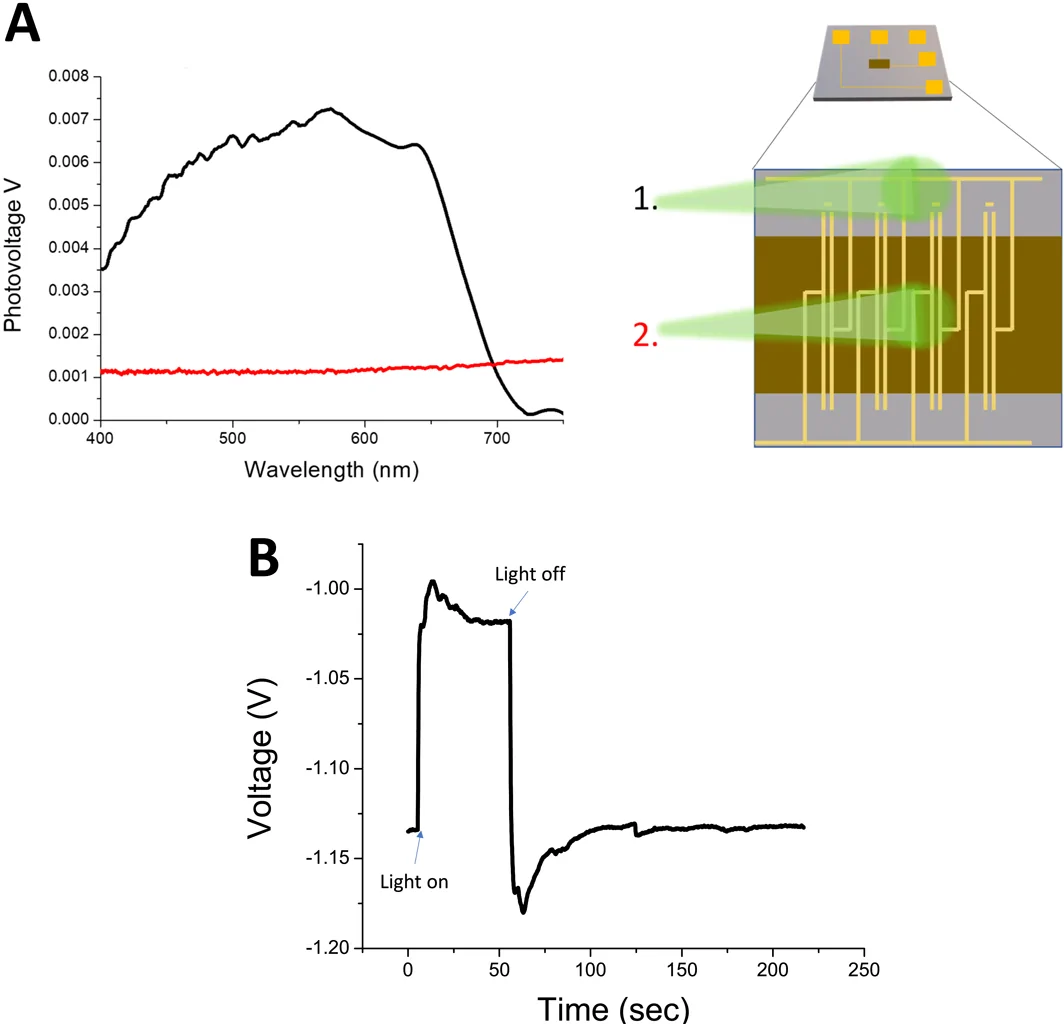
Photovoltage experiments of the plantenna/GO device. (A) The spectral investigation of the response coming from optically initiating the region with the plantenna (region 1) where conversion of photons to plasmons occurs versus region where no such conversion occurs (region two on the device). (B) Measuring of the plantenna/GO potential with time (Light source: 550 nm). Photovoltage was continuously acquired during this process at sampling period of ∼ 0.1 sec. The samples were illuminated using a 300 W Xe light source monochromatized by Newport Corp. MS257 monochromator. To prevent illumination by second order diffractions of the grating, the monochromatized light was further filtered by order‐sorting long pass filters. The photon flux was kept constant throughout the entire spectral acquisition by means of a variable slit operated in closed control loop.

Based on the results presented in Figure 4A we conducted a representative time‐resolved photovoltage measurement, further confirming the plasmonic charging of the GO nanocapacitor. Importantly, Figure 4B demonstrates fast complete charging (up to photovoltage saturation). It is important to note that SPP propagation is much faster than 100msec sampling interval that appears in Figure 4B. What we aim to claim in Figure 4B is that one may see the asymmetry between the temporal response due to photonic related effects that might be associated with plasmonic processes, and those that are associated with electrical/thermal processes. One can see that the rise/fall time response due to illumination is faster than 100msec (sampling time) and it is also much faster than the electrical/thermal temporal response occurring right after the illumination was turned OFF (time window of 60–110 sec in the figure), that is upon turning OFF the light irradiation, the photovoltage decayed back to the baseline signal in thermal based processes (which are slower).

Note that indeed the spectral measurements we performed in our experiments (Figure 4A) do not include the full spectral range matched to the one of solar radiation (Light source: 550 nm). The reason is due to the characterization equipment we used in the lab which involved measurement performed in the lab and generation of excitation by using a tunable light source rather than real solar radiation. However, one of the advantages that our novel technology may offer is that the operability is based upon conversion of photons to plasmons rather than the absorption of photons as happens in semiconducting junctions of photovoltaic (PV) cells. Thus, it is very simple to design the nano antennas that will perform efficient conversion of photons to plasmons in wider spectral range such that the obtainable spectral range will depend on our design rather than on the inherent physical properties of absorption of the material used in the PV cells.

The amplitudes of the photovoltage response to each of the photon energies are given in Figure 5. The difference between the response at peak and the one at the valley is larger when the full device (*plantenna* coated with GO) is measured (difference of 7 mV) and the difference is smaller when no GO coating was applied (difference of 5 mV) while for both the spectral peak as well as for the spectral valley, the full device response is more than 1.8 times stronger than in the case of no GO coating. Note that those are only our preliminary insights that were empirically obtained and they should and will be further investigated in our future research.

**FIGURE 5:**
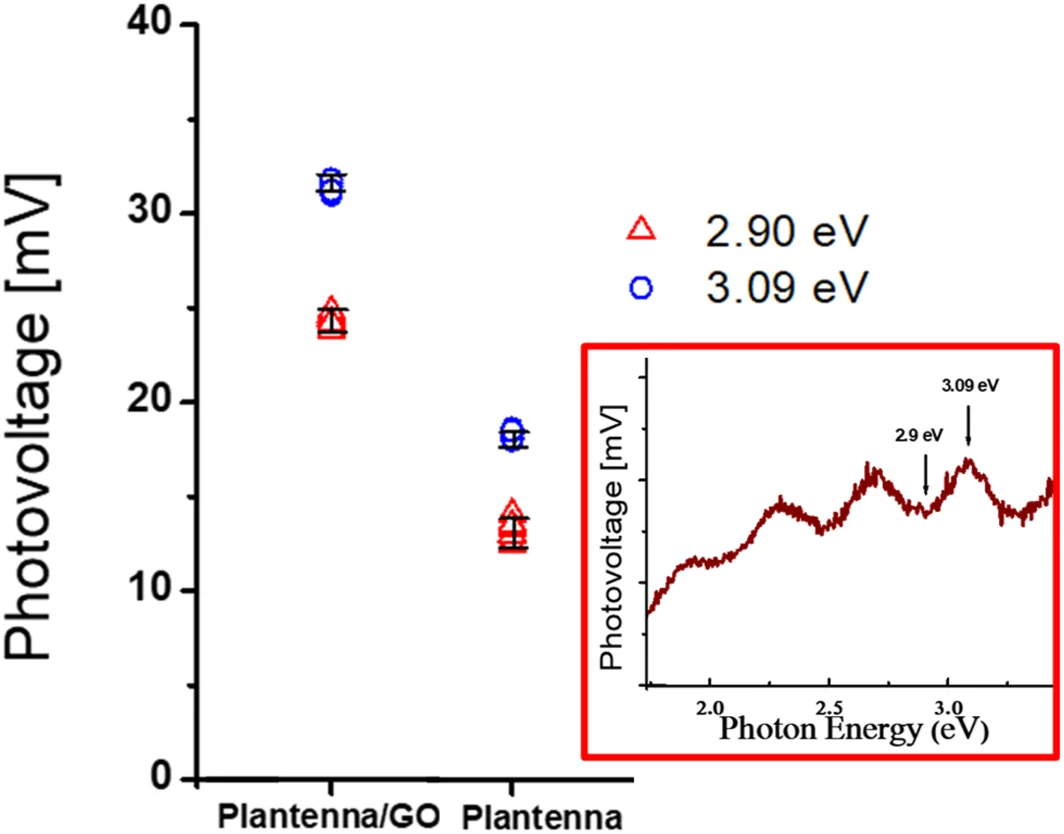
Measurement of the photovoltage response for energy of 2.9 eV (red triangle), and 3.09 eV (blue circle) on devices with and without GO coating.

The experimental data presented introduces a new concept, in which photoinduced plasmons generated in a specially designed *plantenna* can be employed for charging nanocapacitors. Importantly, only the combination of the *plantenna* together with the capacitive layer produced a pronounced photovoltage response being much stronger in respect to no GO coating (e.g., see around the operation wavelength of 400 nm). This response of photovoltage generation due to charging and later on discharging of the nanocapacitor was also validated experimentally both in the time domain (Figure 4) as well as in the spectral domain (e.g., Figure 3).

The electrochemical behavior of the graphene oxide electrode was first investigated by cyclic voltammetry. As shown in Figure 6A, the CV curves exhibit a quasi‐rectangular shape throughout the investigated scan‐rate range, which is characteristic of pseudocapacitive materials. The preservation of this shape even at high scan rates of up to 200 mV s^−1^ indicates rapid charge‐transfer kinetics and efficient electrolyte ion transport within the porous graphene oxide framework. Furthermore, the increasing enclosed area of the CV curves with scan rate suggests enhanced charge‐storage capability while maintaining good electrochemical reversibility.

**FIGURE 6:**
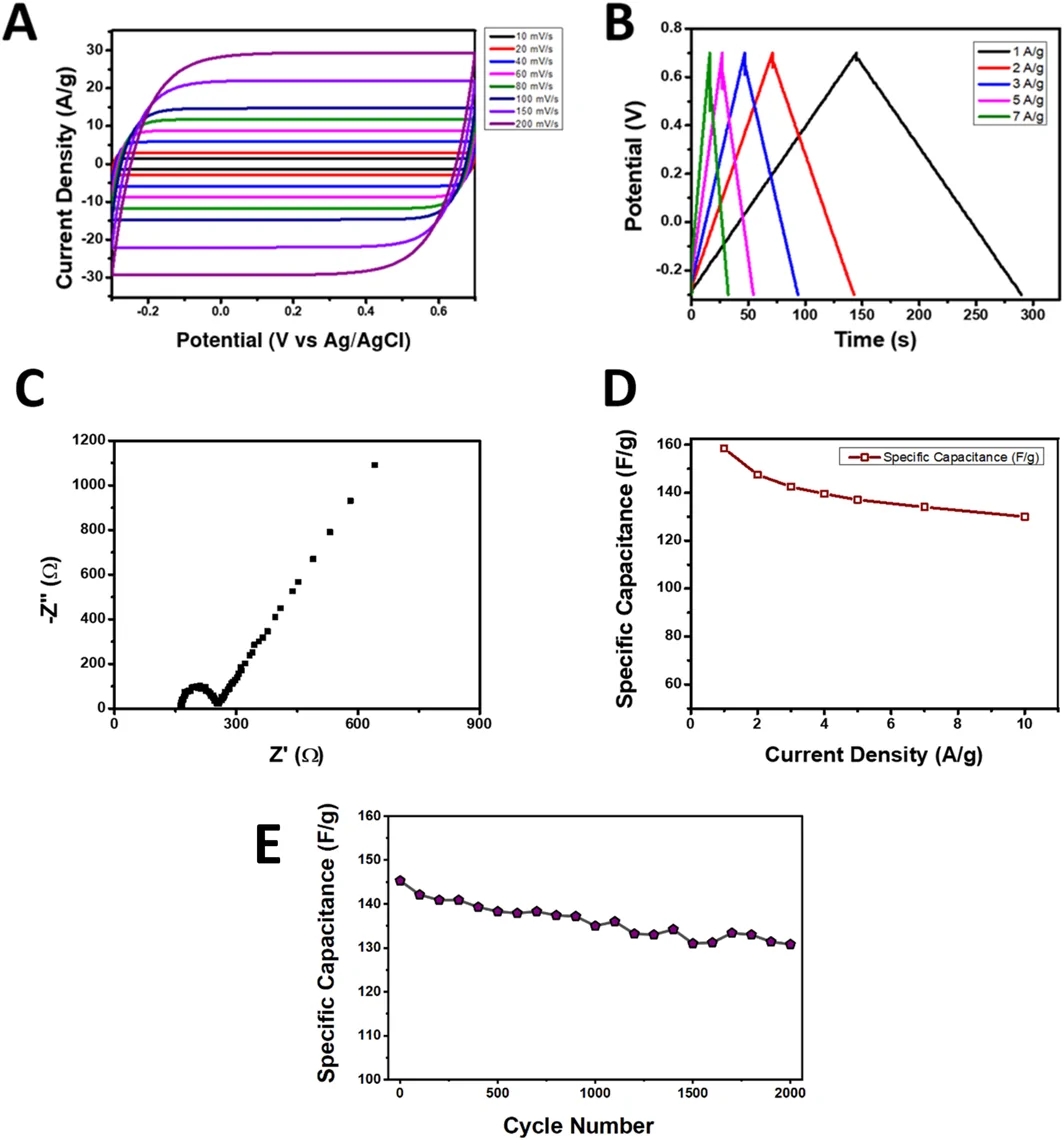
Electrochemical performance of the graphene oxide (GO) electrode. Electrochemical characterization of the graphene oxide (GO) electrode. (A) Cyclic voltammetry (CV) curves recorded at scan rates of 10, 20, 40, 60, 80, 100, 150, and 200 mV s^−1^. (B) Galvanostatic charge‐discharge (GCD) curves measured at current densities of 1, 2, 3, 5 and 7 A g^−1^. (C) Electrochemical impedance spectroscopy (EIS). Nyquist plot of the GO electrode measured over the frequency range of 0.01 Hz–100 kHz. (D) Specific capacitance values calculated from the GCD measurements as a function of current density. (E) Cycling stability of the as‐prepared electrode at a current density of 3 A g^−1^.

Galvanostatic charge‐discharge measurements were subsequently employed to quantify the charge‐storage performance of the material. The GCD profiles exhibit nearly symmetrical quasi‐triangular shapes across all investigated current densities (Figure 6B), indicating highly reversible charging and discharging processes with minimal polarization losses. The specific capacitance values calculated from the discharge curves were 158.6, 147.6, 142.3, 136.9, and 133.7 F g^−1^ at current densities of 1, 2, 3, 5, and 7 A g^−1^, respectively. Although a gradual decrease in capacitance is observed at higher current densities, the electrode retains more than 82% of its initial capacitance when the current density increases tenfold, demonstrating favorable rate capability. This behavior is due to the high specific surface area (∼ 1500 m^2^ g^−1^) and the hierarchical pore structure of the graphene oxide sheets, which facilitate efficient electrolyte diffusion and charge accumulation at the electrode‐electrolyte interface.

Electrochemical impedance spectroscopy was employed to further evaluate the charge transfer and ion diffusion characteristics of the electrode (Figure 6C). The impedance response indicates efficient ion transport and favorable electrolyte penetration throughout the porous graphene oxide network. The observed behavior is consistent with the high surface area and interconnected pore structure of the material, which facilitate rapid ion diffusion and contribute to the overall capacitive performance. Figure 6D summarizes the rate capability of the graphene oxide electrode, showing a gradual decrease in specific capacitance with increasing current density. Nevertheless, the electrode maintained a substantial fraction of its capacitance even at higher current densities, indicating favorable rate performance. The long‐term electrochemical stability was further evaluated through repeated GCD cycling at a current density of 3 A g^−1^ (Figure 6E), with the specific capacitance values presented at intervals of 100 cycles. After 2000 charge–discharge cycles, the electrode retained 92.6% of its initial capacitance. This high capacitance retention demonstrates the good cycling stability and electrochemical durability of the graphene oxide electrode.

To further clarify the practical energy‐storage characteristics of the *plantenna*/GO device, additional quantitative electrostatic characterization was performed. The capacitance of the fabricated devices was measured under dark and illuminated conditions, revealing capacitance values of approximately 5 and 7 nF, respectively, for the *plantenna*/GO configuration, compared to approximately 0.8 nF for the uncoated control device. These results support the contribution of the GO dielectric layer to charge accumulation and storage behavior.

The stored charge was estimated according to (*Q* = CV), yielding charge‐storage values of approximately 9.8 × 10^−10^ C under illumination at 550 nm. The corresponding stored electrostatic energy, calculated using ($E=CV^{2}/2)$ where (*E*) is the stored energy, (*C*) is the measured capacitance of the device, and (*V*) is the experimentally measured photovoltage under illumination, was determined to be approximately 6.9 × 10^−11^ J. These values confirm that the observed photovoltage response is associated with measurable capacitive energy storage rather than solely transient surface photovoltage effects.

Time‐resolved charging and discharging measurements were additionally performed to evaluate the dynamic response of the device. Upon illumination, the photovoltage increased rapidly and reached saturation, while after termination of illumination the signal decayed with a characteristic discharge time constant of approximately 12–15 s whose profiles could be fitted using standard RC‐type exponential behavior, consistent with capacitive discharging mechanisms.

To evaluate device reproducibility, measurements were performed on two independently fabricated chips, each containing two individual *plantenna*/GO devices. The devices exhibited consistent responses, with average peak photovoltage values of approximately 140 ± 12 mV and capacitance variation below 15%. These reproducibility measurements demonstrate the robustness of the fabrication process and support the reliability of the observed plasmon‐mediated charging behavior. Collectively, these additional quantitative analyses further support the interpretation that the *plantenna*/GO architecture operates as a photoinduced plasmon‐mediated nano‐capacitive energy‐storage platform.

The important impact resulting from our research is that the photo generated plasmons were able to energetically charge the electric nanocapacitor despite the fact that plasmons are currents at “optical” frequencies. This was probably due to the inherent nano structure of the designed capacitor. Another important impact is that the photonic energy was converted to electronic one and stored in the electric nanocapacitor. As a result, we were able to produce a preliminary surface device that efficiently converts and stores photonic (solar) energy.

## 4 Conclusions

In this paper we presented a preliminary validation of a novel concept of photo plasmonic voltaic energy storage captured in special nanocapacitor. Overall, the photoinduced plasmon‐mediated capacitor concept may constitute a preliminary basis for innovative “plasmonic solar cells” which could in the future be manufactured from inexpensive plasmonic generating metals coated with readily available dielectric materials. The important advantage of the proposed configuration is that it is a surface fabricated device and does not require volumetric photonic energy conversion via its absorption in the relevant semiconducting material.

Although the proposed concept has a large potential, it is also important to note that the conversion of light into plasmons as well as guiding the plasmonic mode, have large losses that reduce the efficiency in which the photonic energy is converted into the energy stored in the capacitor. The *plantenna* devices we use in this paper were previously designed and characterized [1, 2] and they have relatively improved light to plasmons conversion efficiency in respect to for example Yagi antennas‐based nano‐devices, but nevertheless, the overall conversion efficiency is still relatively low.

## Author Contributions

**Nitzan Shauloff:** investigation, validation, methodology. **Yury Turkulets:** investigation, methodology, validation. **Ilan Shalish:** validation. **Yossi Abulafia:** validation. **Zeev Zalevsky:** conceptualization, writing – original draft, writing – review and editing, methodology. **Raz Jelinek:** conceptualization, methodology, writing – review and editing.

## Funding

This work was supported by the Ministry of Energy, Israel (Grant 218‐11‐035).

## Conflicts of Interest

The authors declare no conflicts of interest.

## Data Availability

The data that support the findings of this study are available from the corresponding author upon reasonable request.
